# Crystal structure of SARS-CoV-2 main protease in complex with protease inhibitor PF-07321332

**DOI:** 10.1007/s13238-021-00883-2

**Published:** 2021-10-22

**Authors:** Yao Zhao, Chao Fang, Qi Zhang, Ruxue Zhang, Xiangbo Zhao, Yinkai Duan, Haofeng Wang, Yan Zhu, Lu Feng, Jinyi Zhao, Maolin Shao, Xiuna Yang, Leike Zhang, Chao Peng, Kailin Yang, Dawei Ma, Zihe Rao, Haitao Yang

**Affiliations:** 1 Shanghai Institute for Advanced Immunochemical Studies and School of Life Science and Technology, ShanghaiTech University, Shanghai, 201210 China; 2 State Key Laboratory of Bioorganic & Natural Products Chemistry, Center for Excellence in Molecular Synthesis, Shanghai Institute of Organic Chemistry, University of Chinese Academy of Sciences, Chinese Academy of Sciences, Shanghai, 200032 China; 3 CAS Center for Excellence in Molecular Cell Science, Shanghai Institute of Biochemistry and Cell Biology, Chinese Academy of Sciences, Shanghai, 200031 China; 4 University of Chinese Academy of Sciences, Beijing, 100049 China; 5 State Key Laboratory of Virology, Wuhan Institute of Virology, Center for Biosafety Mega-Science, Chinese Academy of Sciences, Wuhan, 430071 China; 6 National Facility for Protein Science in Shanghai, Zhangjiang Lab, Shanghai Advanced Research Institute, Chinese Academy of Sciences, Shanghai, 201210 China; 7 Taussig Cancer Center, Cleveland Clinic, Cleveland, OH 44195 USA; 8 Laboratory of Structural Biology, School of Life Sciences and School of Medicine, Tsinghua University, Beijing, 100091 China; 9 State Key Laboratory of Medicinal Chemical Biology, Frontiers Science Center for Cell Response, College of Life Sciences, Nankai University, and Tianjin Key Laboratory of Protein Sciences, Tianjin, 300071 China

**Dear Editor,**

Since December 2019, the pandemic of coronavirus disease 2019 (COVID-19) has taken a heavy toll on global health, creating an urgent need to develop effective strategies for prevention and treatment. The etiological agent, known as severe acute respiratory syndrome coronavirus 2 (SARS-CoV-2), has infected nearly 229.2 million people worldwide with more than 4.7 million deaths as of September 15, 2021. Older age and preexisting health conditions are associated with worse clinical prognosis including higher mortality rates (Zhou et al., 2020). The global race to combat this pandemic has led to rapid deployment of numerous effective vaccines against SARS-CoV-2 (Tregoning et al., 2021). However, the emergence of viral variants, including the Delta variant (B.1.617.2), compromised vaccine effectiveness with resurgence of SARS-CoV-2 infection among highly vaccinated population (Keehner et al., 2021). Therefore, development of therapeutics against the more conserved viral targets would be essential to contain the spread of COVID-19 and reduce mortality.

SARS-CoV-2 is a positive-sense, single-stranded RNA virus, and its genomic sequence shares approximately 80% identity to that of SARS-CoV, the pathogen that caused the SARS outbreak in 2003 (Lu et al., 2020). The replication of SARS-CoV-2 relies on two polyproteins pp1a and pp1ab, which are encoded by the 5’ two-thirds of the viral genome (Cui et al., 2020). Both pp1a and pp1ab are co-translationally cleaved into 16 mature nonstructural proteins (nsps) by the two viral cysteine proteases, the main protease (M^pro^, also called 3C like protease, 3CLpro) and the papain like protease (PLpro) (Jin et al., 2020; Zhao et al., 2021). SARS-CoV-2 M^pro^ processes the two polyproteins through at least 11 cleavage sites. These nsps, including helicase, RNA-dependent RNA polymerase (RdRp), and methyltransferase and so on, then assemble into the replication-transcription complex to promote viral replication (Cui et al., 2020). From an evolutionary perspective, the amino acid sequence and 3-dimensional structure of M^pro^ are highly conserved throughout the subfamily *Coronavirinae*, providing a strong mechanistic basis for designing therapeutics to address the recent emergence of SARS-CoV-2 variants with concerns for immune evasion (Wang et al., 2016; Jin et al., 2020). Examination of M^pro^ sequences across known SARS-CoV-2 variants revealed low mutation frequency (Table S1 and Fig. S1). In addition, the essential role of M^pro^ in viral life cycle and lack of its homolog in the human beings make it an attractive target for drug development against SARS-CoV-2 (Yang et al., 2005).

Using a platform consisted of structure-assisted drug design, virtual drug screening, and high-throughput screening, we previously discovered several lead compounds including ebselen and carmofur that potently inhibit SARS-CoV-2 M^pro^ (Jin et al., 2020). Given the genomic and structural similarity between SARS-CoV and SARS-CoV-2, several inhibitors rationally designed against SARS-CoV M^pro^, such as N3 and PF-00835231, also demonstrated potent inhibitory activity against SARS-CoV-2 M^pro^ (Hoffman et al., 2020; Jin et al., 2020). However, the low oral bioavailability of PF-00835231 limits its clinical potential, which has been improved using its phosphate prodrug, PF-07304814 (Boras et al., 2021). PF-07304814 is highly soluble that permits dosing with continuous infusion. Recently, Pfizer announced the development of the second-generation orally available SARS-CoV-2 M^pro^ inhibitor, PF-07321332, which has been evaluated in a dose escalation phase 1 study for safety (NCT04756531) (Vandyck and Deval, 2021). The phase 2/3 clinical trials (NCT04960202 and NCT05011513) to evaluate the safety and efficacy of PF-07321332/ritonavir combination among non-hospitalized adult patients with COVID-19 are currently in progress. Co-administration of low dose ritonavir is expected to slow down the metabolic degradation of PF-07321332 in the body.

To elaborate its inhibitory mechanism, we determined the crystal structure of SARS-CoV-2 M^pro^ in complex with PF-07321332 at 1.6 Å resolution (Table S2). PF-07321332 is a reversible covalent inhibitor carrying a nitrile warhead that targets SARS-CoV-2 M^pro^ (Fig. 1A). The overall structure of SARS-CoV-2 M^pro^ is a functional dimer, consistent with our previous findings (Yang et al., 2005; Wang et al., 2016; Jin et al., 2020). All of the residues (1–306) could be traced according to the electron density map. Each protomer contains three domains (Fig. 1B). Domain I (residues 10–99) and domain II (residues 100–184) are primarily consisted of β-barrel folds. Domain III (residues 201–303) contains five α-helices, and it is relatively independent and connected to domain II through a long loop region (residues 185–200). The substrate-binding pocket that features a non-canonical dyad of cysteine 145 and histidine 41 (C145 and H41) lies in the cleft between domains I and II (Fig. 1B and C).

**Figure 1:**
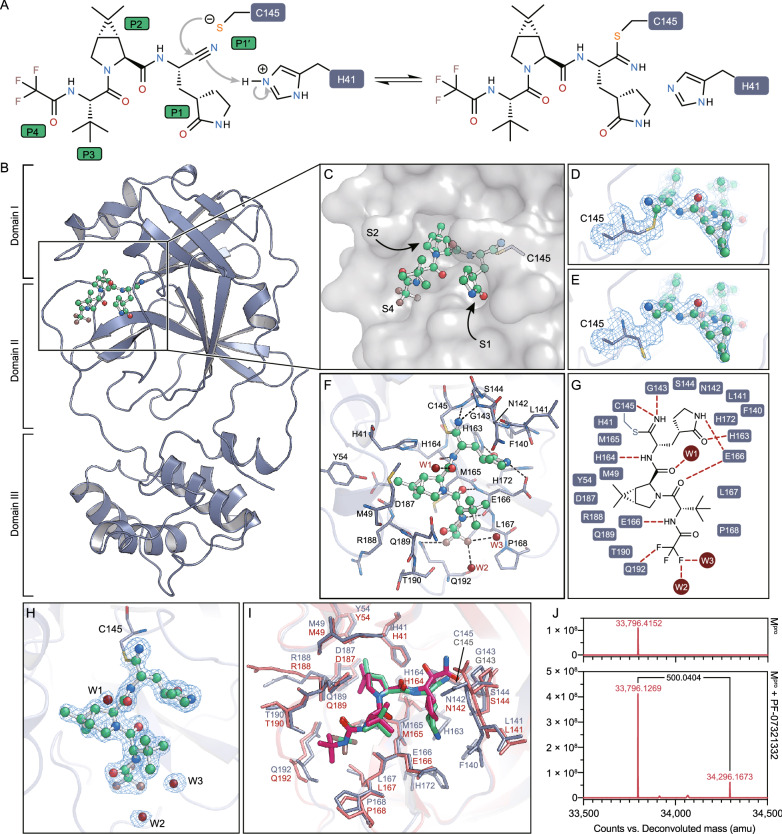
**The structure of SARS-CoV-2 M**^**pro**^** in complex with PF-07321332.** (A) A putative inhibition mechanism of PF-07321332. The catalytic C145 and H41 of SARS-CoV-2 M^pro^ are highlighted with dark blue squares. P1’, P1, P2 and P4 are highlighted with green squares. (B) The overall structure of a SARS-CoV-2 M^pro^ protomer. The three domains of M^pro^ are labeled. The substrate-binding pocket is located within the black box. PF-07321332 is shown as ball-and-stick model with the carbon atoms in bright green, oxygen atoms in bright red, and nitrogen atoms in blue, and fluorine atom in brown. (C) The zoom-in view of the substrate-binding pocket. PF-07321332 forms a covalent bond to C145. Three substrate-binding subsites (S1, S2 and S4) are labeled. (D) The Sγ atom of the catalytic C145 forms a 1.8-Å C-S covalent bond with the nitrile carbon of PF-07321332. The 2*F*_*o*_-*F*_*c*_ density map contoured at 1.2σ is shown in the light blue mesh. (E) The catalytic C145 in another state where it does not form a covalent bond with PF-07321332. The 2*F*_*o*_-*F*_*c*_ density map contoured at 1.2σ is shown in the light blue mesh. (F) The detailed binding model in the SARS-CoV-2 M^pro^-PF07321332 complex structure. The residues that participate in inhibitor binding are shown as sticks. (G) Schematic diagram of the interactions between PF-07321332 and SARS-CoV-2 M^pro^. (H) The polder density map of PF-07321332, which is colored as the light blue mesh and contoured at 3.0 σ. (I) The comparison of SARS-CoV-2 M^pro^-PF07321332 complex structure with that of SARS-CoV-2 M^pro^-boceprevir. (J) The molecular weights of apo SARS-CoV-2 M^pro^ and PF-07321332 treated M^pro^ determined from tandem mass spectrometry. The mass shift (∆m) of the protein is labeled

The unambiguous electron density map demonstrates that the nitrile carbon of PF-07321332 is linked to the Sγ atom of C145 through a standard 1.8-Å C-S covalent bond (Fig. 1D and H). This suggests that the thiol group of catalytic C145 attacks the electrophilic nitrile group at P1′ site of PF-07321332, resulting in the formation of a thioimidate adduct (Fig. 1A). This result is also supported by tandem mass spectrometry analysis, which revealed the presence of a mass shift of 500 Dalton (Fig. 1J). The reversibility of the covalent bond was supported by the dual conformations of the catalytic cysteine based on the electron density map, with the thiol group of cysteine not directly bonded to nitrile group of the inhibitor in the alternative conformation (Fig. 1E). The imine nitrogen of the thioimidate moiety occupies the oxyanion hole that is stabilized by the backbone NH of G143 and C145 through two hydrogen bonds (Fig. 1F and G). The classical (S)-γ-lactam ring at P1 position fits into the S1 subsite in a similar pattern to several other rationally designed inhibitors such as N3, 11a, and 11b (Dai et al., 2020; Jin et al., 2020). The hydrogen bond between the oxygen atom of the lactam ring and Nε2 atom of H163 stabilizes the inhibitor (Fig. 1F and G). Besides, the Oε1 atom of E166 also interacts with the NH group of the lactam ring through a hydrogen bond (Fig. 1F and G). The amide nitrogen at P1 site forms a hydrogen bond with the main-chain carbonyl oxygen of H164 and the amide oxygen forms another hydrogen bond with an ordered water molecule W1 (Fig. 1F and G).

Similar to the structure of SARS-CoV-2 M^pro^ in complex with boceprevir (Fu et al., 2020), the S2 subsite is occupied by the rigid dimethylcyclopropylproline (DMCP) group (Fig. 1C, F, and I). This hydrophobic group is surrounded by the side chains of H41, M49, Y54, M165 and D189, and the main chains of D187 and R188, resulting in extensive hydrophobic interactions (Fig. 1F and G). The hydrophobic tert-butyl group at P3 was exposed to the solvent with limited interactions with M^pro^ (Fig. 1C). The trifluoroacetyl group at P4 site is accommodated by the S4 sub-pocket. The amide nitrogen at P4 site forms a hydrogen bond with the main chain carbonyl oxygen of E166 (Fig. 1F and 1G). Compared with SARS-CoV-2 M^pro^-boceprevir complex structure, the trifluoromethyl group of PF-07321332 forms additional hydrogen bonds by interacting with the Nε2 atom Q192 and two ordered water molecules W2 and W3, which provides stronger interactions to anchor the P4 site (Fig. 1F–H). Therefore, in addition to the S-C covalent bond connecting the nitrile carbon with the catalytic C145, the inhibitor PF-07321332 is further stabilized through a network of hydrogen bonds and hydrophobic interactions, which enhance its binding to the active site of SARS-CoV-2 M^pro^.

Protease inhibitors have been proven to be an effective pharmaceutical strategy to combat both hepatitis C virus (HCV) and human immunodeficiency virus (HIV) infections in the clinic. Peptidomimetic inhibitors, such as boceprevir and telaprevir, carry a warhead of α-ketoamide and form a reversible covalent bond with the catalytic serine residue at the active site of HCV NS3/4A protease (Manns and von Hahn, 2013). Such mechanism-based inactivation of viral protease through forming a covalent bond enhances the selectivity to the viral target. We previously demonstrated that N3, a peptidomimetic compound with a Michael acceptor that forms an irreversible covalent bond with the catalytic cysteine, exhibited broad-spectrum inhibition of the M^pro^ across both human and animal CoVs, including the SARS-CoV-2 (Yang et al., 2005; Wang et al., 2016; Jin et al., 2020). The complex structure of SARS-CoV-2 M^pro^ with PF-07321332 demonstrates the efficacy of the warhead to form a covalent bond with the cysteine residue at the active site. The lactam ring, DMCP group, and trifluoroacetyl group enhance the binding of the inhibitor at the S1, S2, and S4 subsites of the substrate-binding pocket. Similar to PF-00835231 and N3 (Yang et al., 2005; Hoffman et al., 2020), PF-07321332 may exhibit wide spectrum inhibition against M^pro^ beyond SARS-CoV-2, highlighting the potential to be used for other human CoVs.

In conclusion, the 1.6-Å crystal structure of SARS-CoV-2 M^pro^ in complex with the protease inhibitor PF-07321332 reveals critical insight for pharmaceutical development. With the emergence of SARS-CoV-2 variants with immune evasion, therapeutics targeting a conserved but essential viral target among the variants may synergize the COVID-19 vaccines. The improved oral bioavailability would enhance the clinical utility of PF-07321332 as a therapeutic to prevent progression among early-stage non-hospitalized patients or as a prophylaxis in the pre- and post-exposure setting. Further optimization of such covalent peptidomimetic inhibitors would generate potent drugs to contain the current COVID-19 pandemic and increase public health preparedness for potential future pandemic.

## Supplementary Information

Below is the link to the electronic supplementary material.

Supplementary file1 (PDF 403 kb)
