# Supplementary material for: Crystal structure of SARS-CoV-2 main protease in complex with protease inhibitor PF-07321332
Source: Protein Cell. 2021 Oct 22;13(9):689–93. doi: 10.1007/s13238-021-00883-2 (PMC8533666; doi:10.1007/s13238-021-00883-2)
Supplement: Supplementary file 1 — Supplementary file1 (PDF 403 kb) [file 13238_2021_883_MOESM1_ESM.pdf]

SUPPLEMENTAL INFORMATION

Crystal structure of SARS-CoV-2 main protease in complex with protease inhibitor PF-07321332

Yao Zhao[1†], Chao Fang[2†], Qi Zhang[1,3,4†], Ruxue Zhang[5], Xiangbo Zhao[2], Yinkai Duan[1], Haofeng Wang[1], Yan Zhu[1], Lu Feng[1], Jinyi Zhao[1], Maolin Shao[1], Xiuna Yang[1], Leike Zhang[5], Chao Peng[6], Kailin Yang[7], Dawei Ma[2*], Zihe Rao[1,8,9*], Haitao Yang[1*]

[1]Shanghai Institute for Advanced Immunochemical Studies and School of Life Science and Technology, ShanghaiTech University, Shanghai 201210, China

[2]State Key Laboratory of Bioorganic & Natural Products Chemistry, Center for Excellence in Molecular Synthesis, Shanghai Institute of Organic Chemistry, University of Chinese Academy of Sciences, Chinese Academy of Sciences, Shanghai 200032, China

[3]CAS Center for Excellence in Molecular Cell Science, Shanghai Institute of Biochemistry and Cell Biology, Chinese Academy of Sciences, Shanghai 200031, China

[4]University of Chinese Academy of Sciences, Beijing 100049, China

[5]State Key Laboratory of Virology, Wuhan Institute of Virology, Center for Biosafety Mega-Science, Chinese Academy of Sciences, Wuhan 430071, China

[6]National Facility for Protein Science in Shanghai, Zhangjiang Lab, Shanghai Advanced Research Institute, Chinese Academy of Sciences, Shanghai 201210, China

[7]Taussig Cancer Center, Cleveland Clinic, Cleveland, OH 44195, USA

[8]Laboratory of Structural Biology, School of Life Sciences and School of Medicine, Tsinghua University, Beijing 100091, China

[9]State Key Laboratory of Medicinal Chemical Biology, Frontiers Science Center for Cell Response, College of Life Sciences, Nankai University, and Tianjin Key Laboratory of Protein Sciences, Tianjin 300071, China

[†]These authors contributed equally: Yao Zhao, Chao Fang, and Qi Zhang.

*Correspondence: madw@sioc.ac.cn (D.M.), raozh@mail.tsinghua.edu.cn (Z.R.), yanght@shanghaitech.edu.cn (H.Y.).

MATERIALS AND METHODS

Cloning, protein expression, and purification of SARS-CoV-2 M^{pro}

The cell cultures were grown and the protein was expressed according to a previous report (Jin et al., 2020). The cell pellets were resuspended in lysis buffer (20mM Tris-HCl pH 8.0, 150 mM NaCl, 5% Glycerol), lysed by high-pressure homogenization, and then centrifuged at 25,000g for 30 min. The supernatant was loaded onto Ni-NTA affinity column (Qiagen, Germany), and washed by the lysis buffer containing 20 mM imidazole. The His-tagged M^{pro} was eluted by lysis buffer containing 300 mM imidazole. The imidazole was then removed through desalting. Human rhinovirus 3C protease was added to remove the C-terminal His tag. SARS-CoV-2 M^{pro} was further purified by ion exchange chromatography. The purified M^{pro} was transferred to 10 mM Tris-HCl pH 8.0 through desalting and stored at -80 degrees until needed.

Crystallization, data collection, and structure determination

PF-07321332 is synthesized by Prof. Ma's Lab. SARS-CoV-2 M^{pro} (6 mg/ml) was incubated with 1 mM PF-07321332 for 1 hour at room temperature and the complex was crystallized by hanging drop vapor diffusion method at 20 °C. The best crystals were grown using a well buffer containing 0.1 M MES pH 6.0, 5% polyethylene glycol (PEG) 6000, and 3% DMSO. The cryo-protectant solution was the reservoir but with 20% glycerol added.

X-ray data were collected on beamline BL19U1 at Shanghai Synchrotron Radiation Facility (SSRF) at 100 K and at a wavelength of 0.97852 Å using a Pilatus3 6M image plate detector. Data integration and scaling were performed using the program XDS (Kabsch, 2010). The structure was determined by molecular replacement (MR) with the PHASER (McCoy et al., 2007) and Phenix 1.19.2 (Liebschner et al., 2019) using the SARS-CoV-2 M^{pro} (PDB accession number: 6LU7) as a search template. The model from MR was subsequently subjected to iterative cycles of manual model adjustment with Coot 0.8 (Emsley et al., 2010) and refinement was completed with Phenix REFINE (Afonine et al., 2012). The inhibitor PF-07321332 was built according to the omit map. The phasing and refinement statistics are summarized in Table S1. Coordinates and structure factors have been deposited in PDB with the accession number 7VH8.

Intact protein analysis

1 μL PF-07321332 (10mM in DMSO) was add into 50uL of the proteins (1 mg/mL). The mixtures were kept in room temperature for 30 min. Liquid chromatography-mass spectrometry (LC-MS) analyses were performed in positive-ion mode with an Agilent 6550 quadrupole-time-of-flight

(QTOF) mass spectrometer (Santa Clara, CA) coupled with an Agilent 1260 high-performance liquid chromatograph (HPLC; Santa Clara, CA) for detecting the molecular weight of intact proteins. The samples were eluted from a Phenomenex Jupiter C4 300Å LC Column (2×150 mm, 5 μm) over a 15 min gradient from 5% to 100% acetonitrile containing 0.1% formic acid at a flow rate of 0.5 mL/min. The acquisition method in positive-ion mode with Dual Agilent Jet Stream electrospray voltage used a capillary temperature of 250 °C, a fragmentor of 175 V, a capillary voltage of 3000 V. Mass deconvolution was performed using Agilent MassHunter Qualitative Analysis B.06.00 software with BioConfirm Workflow.

Tandem mass spectrometry analysis

The samples were precipitated and resolved with 8 M urea, and then digested for 16 h at 25 °C by chymotrypsin at an enzyme-to-substrate ratio of 1:50 (wt/wt). The digested peptides were desalted and loaded onto a homemade 30 cm-long pulled-tip analytical column (ReproSil-Pur C18 AQ 1.9 μm particle size, Dr. Maisch GmbH, 75 μm ID× 360 μm OD) connected to an Easy-nLC1200 UHPLC (Thermo Scientific) for mass spectrometry analysis. The elution gradient and mobile phase constitution used for peptide separation were as follows: 0-1 min, 5%-8% B; 1-114 min, 8-35% B; 115-116 min, 35-50% B; 116-120min, 60-100% B (mobile phase A: 0.1% Formic Acid in Water and mobile phase B: 0.1% formic acid in 80% Acetonitrile) at a flow rate of 300 nL /min. Peptides eluted from the LC column were directly electro-sprayed into the mass spectrometer with the application of a distal 1.8-kV spray voltage. Survey full-scan MS spectra (from m/z 300–1500) were acquired in the Orbitrap analyzer (Eclipse) with resolution r =120,000 at m/z 400. The dynamic exclusion time was set at 30 seconds. One acquisition cycle includes one full-scan MS spectrum followed by top MS/MS events in the cycle time setting at 3 s, sequentially generated on the most intense ions selected from the full MS spectrum at a 30% normalized collision energy. The acquired MS/MS data were analyzed UniProtKB *E. coli* database (database released on Nov. 11, 2016) containing nsp5 using Protein Discoverer 2.4. In order to accurately estimate peptide probabilities and false discovery rates (FDR), we used a decoy database containing the reversed sequences of all the proteins appended to the target database. FDR was set at 0.01. Mass tolerance for precursor ions was set at 20 ppm. Chymotrypsin was defined as cleavage enzyme and the maximal number of missed cleavage sites was set at 4. Protein N-terminus acetylation, methionine oxidation and compounds covalent bindings were set as variable modifications. The modified peptides were manually checked and labeled.

The synthesis of PF-07321332

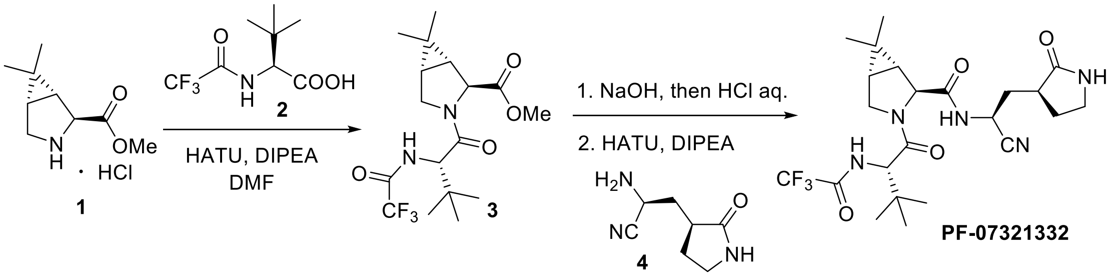

The starting material (amine salt 1) were prepared according to the literature procedure (Venkatraman et al., 2006).

To a solution of amine salt **1** (400 mg, 1.9 mmol) in 10 mL DMF was added HATU (1.1 g, 2.9 mmol), amino acid **2** (633 mg, 2.9 mmol), and DIPEA (1.6 mL, 9.7 mmol), the resulting solution was stirred at room temperature 1 h. The reaction mixture was diluted with water and then extracted with EtOAc. The combined organic phase was concentrated in vacuo and purified by silica gel column chromatography (eluting with EtOAc/hexane 1:10 to 1:5) to obtain methyl ester **3** (589 mg, 80% yield). ESI: m/z 379.0 $[M+H]^+$.

To a solution of methyl ester **3** (40 mg, 0.11 mmol) in toluene/MeOH (10:1, 1.1 ml) was added 47% sodium hydroxide solution (40 μL) dropwise, the resulting mixture was stirred for 1 h under room temperature. The reaction solution was diluted with 1M HCl (aq.) and then extracted with ethyl acetate. The combined organic extracts were concentrated to afford the crude carboxylic acid. A solution of this intermediate in 2 mL DMF was treated with HATU (60 mg, 0.14 mmol), amine **4** (17 mg, 0.12 mmol), DIPEA (100 μL, 0.53 mmol), and stirred at room temperature for 5 h. The reaction mixture was added into water (50 mL) and extracted with EtOAc, the combined organic phase was concentrated and purified by silica gel column chromatography (eluting with EtOAc/hexane 1:5 to 1:1) to yield the target **PF-07321332** (39 mg, 75% yield). ^{1}H NMR (400 MHz, CDCl3) δ 4.98 – 4.72 (m, 1H), 4.63 – 4.48 (m, 1H), 4.33 – 4.20 (m, 1H), 4.18 – 3.96 (m, 1H), 3.84 – 3.58 (m, 1H), 3.47 – 3.24 (m, 2H), 2.64 – 2.29 (m, 3H), 1.93 – 1.76 (m, 2H), 1.52 (dd, J = 10.8, 6.9 Hz, 2H), 1.26 (s, 3H), 1.07 (s, 3H), 1.01 (s, 9H).

REFERENCES:

Afonine, P.V., Grosse-Kunstleve, R.W., Echols, N., Headd, J.J., Moriarty, N.W., Mustyakimov, M., Terwilliger, T.C., Urzhumtsev, A., Zwart, P.H., and Adams, P.D. (2012). Towards automated crystallographic structure refinement with phenix.refine. Acta Crystallogr D Biol Crystallogr 68, 352-367.

Emsley, P., Lohkamp, B., Scott, W.G., and Cowtan, K. (2010). Features and development of Coot. Acta Crystallogr D Biol Crystallogr 66, 486-501.

Jin, Z., Du, X., Xu, Y., Deng, Y., Liu, M., Zhao, Y., Zhang, B., Li, X., Zhang, L., Peng, C.*, et al.* (2020). Structure of M(pro) from SARS-CoV-2 and discovery of its inhibitors. Nature 582, 289-293.

Kabsch, W. (2010). Xds. Acta Crystallogr D Biol Crystallogr 66, 125-132.

Liebschner, D., Afonine, P.V., Baker, M.L., Bunkóczi, G., Chen, V.B., Croll, T.I., Hintze, B., Hung, L.W., Jain, S., McCoy, A.J.*, et al.* (2019). Macromolecular structure determination using X-rays, neutrons and electrons: recent developments in Phenix. Acta Crystallogr D Struct Biol 75, 861-877.

McCoy, A.J., Grosse-Kunstleve, R.W., Adams, P.D., Winn, M.D., Storoni, L.C., and Read, R.J. (2007). Phaser crystallographic software. J Appl Crystallogr 40, 658-674.

Venkatraman, S., Bogen, S.L., Arasappan, A., Bennett, F., Chen, K., Jao, E., Liu, Y.T., Lovey, R., Hendrata, S., Huang, Y.*, et al.* (2006). Discovery of (1R,5S)-N-[3-amino-1-(cyclobutylmethyl)-2,3-dioxopropyl]- 3-[2(S)-[[[(1,1-dimethylethyl)amino]carbonyl]amino]-3,3-dimethyl-1-oxobutyl]- 6,6-dimethyl-3-azabicyclo[3.1.0]hexan-2(S)-carboxamide (SCH 503034), a selective, potent, orally bioavailable hepatitis C virus NS3 protease inhibitor: a potential therapeutic agent for the treatment of hepatitis C infection. J Med Chem 49, 6074-6086.

Table S1. List of M^{pro} mutations among SARS-CoV-2 variants.

WHO Label	Pango Lineage	Mutations of M^{pro}
Alpha	B.1.1.7	-
Beta	B.1.351	K90R
Gamma	P.1	-
Delta	B.1.617.2	-
Eta	B.1.525	-
Iota	B.1.526	-
Kappa	B.1.617.1	-
Lambda	C.37	G15S
Mu	B.1.621	-

Table S2. Data collection and refinement statistics.

	M^{pro}-PF07321332
	PDB code: 7VH8
**Data Collection**	
Space group	$P2_12_12$
Wavelength (Å)	0.97985
Cell dimensions	
a, b, c (Å)	45.341, 63.256, 105.953
a, b, g (°)	90, 90, 90
Resolution (Å)	34.45-1.59 (1.65-1.59)
No. of unique reflections	41812 (4115)
Completeness (%)	99.92 (99.78)
R_{merge}	0.08462 (1.154)
Mean $I/\sigma(I)$	16.67 (1.63)
*CC*1/2	99.8 (85.7)
Redundancy	13.0 (13.4)
Wilson B factors (Å^2)	22.80
**Refinement**	
Resolution (Å)	34.45-1.59
No. of reflections used	41796 (4109)
R_{work} / R_{free}	18.71/19.58
No. atoms	
Protein	2381
Ligand/ion	40
Water	379
B-factors (Å^2)	
Protein	26.77
Ligand/ion	24.23
Water	39.44
R.m.s. deviations	
Bond lengths (Å)	0.020
Bond angles (°)	1.54
Ramachandran plot (%)	
Favored (%)	98.36
Allowed (%)	1.64
Outliers (%)	0

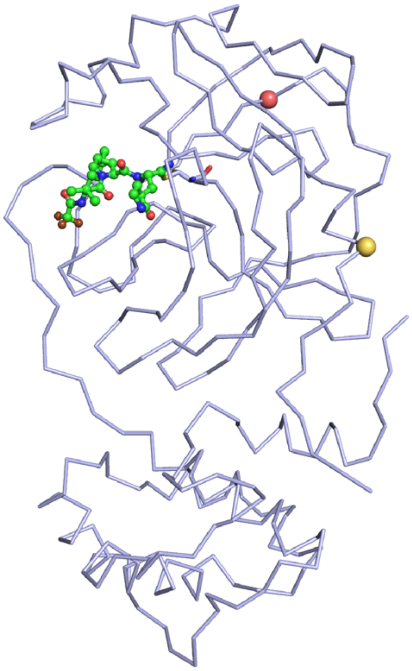

Figure S1. Mapping of the M^{pro} mutations from SARS-CoV-2 variants onto the structure of SARS-CoV-2 M^{pro} in complex with PF-07321332. M^{pro} is shown as light blue ribbon. PF-07321332 is shown as ball-and-stick model with the carbon atoms in bright green, oxygen atoms in bright red, nitrogen atoms in blue, and fluorine atom in brown. Mutation sites are shown as colored spheres. G15S mutantion (from Lambda variant) is colored in yellow; K90R mutantion (from Beta variant) is colored in deep salmon.
